# Potential ameliorative role of *Spirulina platensis* in powdered or extract forms against cyclic heat stress in broiler chickens

**DOI:** 10.1007/s11356-022-19115-z

**Published:** 2022-02-11

**Authors:** Ahmed M. Elbaz, Ayman M. H. Ahmed, Ahmed Abdel-Maqsoud, Aml M. M. Badran, Abdel-Moneim Eid Abdel-Moneim

**Affiliations:** 1 Desert Research Center, Mataria, Cairo, Egypt; 2 Poultry Production Department, Faculty of Agriculture, Ain Shams University, Cairo, Egypt; 3 Poultry Breeding Department, Animal Production Research Institute, Agricultural Research Center, Ministry of Agriculture, Dokki, Giza, Egypt; 4 Biological Applications Department, Nuclear Research Center, Egyptian Atomic Energy Authority, Cairo, 13759 Egypt

**Keywords:** *Spirulina platensis*, Performance, Antioxidant activity, Immune response, High ambient temperature, Broilers

## Abstract

Global warming has become intensified and widespread, threatening the world with causing acute heatwaves that adversely affect poultry production and producers' profitability. *Spirulina platensis* is a precious and promising mitigating strategy to combat the detrimental impacts of heat stress due to its high contents of nutrients and bioactive components. The current study was designed to compare the incorporation impact of *S. platensis* powder or aqueous extract on the growth and physiological responses of heat-stressed broiler chicks. Six hundred 1-day-old Ross 308 male broiler chicks were allocated into five experimental groups with six replicates of 20 chicks each. The control group fed the basal diet without additives, SPP1 and SPP2 groups fed the basal diet with 1 g/kg and 2 g/kg *S. platensis* powder, respectively, while SPE1 and SPE2 groups received 1 ml/L and 2 ml/L *S. platensis* aqueous extract in the drinking water, respectively. All birds were exposed to cyclic heat stress (34 ± 2 °C for 12 h) for three successive days a week from day 10 to day 35. In vitro analysis showed that total phenols, flavonoids, and antioxidant activity of *S. platensis* were remarkably decreased (*P* < 0.001) in the aqueous extract compared to the powder form. Body weight, weight gain, and feed conversion ratio were improved (*P* < 0.001) in all treated groups, while carcass yield and dressing percentage were increased only in SPP1 and SPP2. Feed and water intake and blood biochemical parameters were not affected. Both forms of *S. platensis* enhanced the lipid profile, redox status, and humoral immune response of heat-stressed chicks superior to the powder form. Conclusively, the powder form of *S. platensis* was more effective in enhancing the productivity of broilers and alleviating the negative impacts of heat stress than the aqueous extract form.

## Introduction

Climate change and global warming have become rapid, widespread, and intensified, threatening the world with causing longer warm seasons and more heatwaves. These conditions adversely affect poultry production globally, in particular, in subtropical and tropical countries. Because of the lack of sweat glands, chickens are vulnerable to the elevation in ambient temperature, which causes many physiological disturbances such as suppressed immunocompetence, endocrine disorders, acid-based imbalance, muscle tremors, weakness, and oxidative stress (Bogin et al. 1996; Tao and Xin, 2003). These physiological disorders lead to reduced weight gain and feed consumption, increased water consumption and mortality, and impairment of birds’ overall welfare and productivity (Abdel-Moneim et al. 2021b; Abo Ghanima et al. 2020b; Sakamoto et al. 2020). In addition, the heat stress-induced excessive amount of reactive oxygen species (ROS) contributes to severe tissue injuries and deteriorates the maintenance of homeostasis (Khan et al. 2012; Surai 2007). To combat these detrimental impacts of heat stress, implementing environmental and nutritional mitigation strategies has become mandatory. Lowering stocking densities, increasing the ventilation rate, using different flooring systems, establishing intermittent light schedules, and implementing wet feeding or early feed restriction are among the environmental strategies (Abo Ghanima et al. 2020a; Sahin et al. 2009; Saleh et al. 2021). However, the nutritional strategies are including the use of probiotics (Abd El‐Hack et al. 2020b; Abdel-Latif et al. 2018; Abdel-Moneim et al. 2020b; Abdel-Moneim et al. 2020c; Abou-Kassem et al. 2021), prebiotics (Abd El-Hack et al. 2021; Houshmand et al. 2012), organic acids (Ding et al. 2020; Elbaz et al. 2021), minerals and vitamins (Abd El-Hack et al. 2018; Abdel-Moneim et al. 2021c; Harsini et al. 2012; Khan et al. 2012; Mohamed et al. 2020; Saleh et al. 2020), and herbal extracts and derivatives (Abd El-Hack et al. 2019; Abd El-Hack et al. 2020a, b; Abd El-Moneim and Sabic, 2019; Abd El-Moneim et al. 2019; Sugiharto 2020). Furthermore, phytochemicals, such as polyphenols, have been characterized for their antioxidant capabilities and presented as potential mitigators of heat stress in poultry (Abdel-Latif et al. 2021; Abdel-Moneim et al. 2020d; Hu et al. 2019; Saeed et al. 2018; Saeed et al. 2020).

*Spirulina platensis* (the blue-green algae) is a unicellular microalga that can be grown in fresh and salt aquatic systems and is known as a good source of minerals, proteins, phytopigments, and vitamins (Farag et al. 2016). The high contents of various nutrients in *S. platensis* made it a promising alternative feed additive in poultry diets. Furthermore, *S. platensis* contains numerous bioactive components in high amounts, such as phycocyanins, steroid, saponin, chlorophyll, β-carotene, flavonoid, triterpenoid, and phenolic acids (Agustini et al. 2015). These bioactive compounds have numerous biological activities such as antioxidant, antiviral, anti-inflammatory, hepatoprotective, immunostimulatory, and antimicrobial (Abdel-Daim et al. 2019; Abdel-Moneim et al. 2021a; Abedin and Taha, 2008; Aladaileh et al. 2020). A growing body of literature has attributed these medicinal properties of *S. platensis* to their ability to modulate cytokines and antibodies production, inhibit lipid peroxidation, enhance disease resistance, and scavenge free radicals effectively, thereby improving the productivity and profitability of the poultry industry (Abdel-Daim et al. 2019, 2020; Abdelkhalek et al. 2015; Aladaileh et al. 2020; Evans et al. 2015; Farag et al. 2016). Taken together, these different mechanisms qualify *S. platensis* to be used to mitigate the deleterious impacts of heat stress in poultry (Abdel-Moneim et al. 2021c).

To our knowledge, however, no previous studies have compared the efficacy of *S. platensis* in powder and aqueous extract forms in alleviating the deleterious impacts of heat stress in broiler chicks. Therefore, the current study was designed to compare the incorporation effect of *S. platensis* in its powder and aqueous extract forms on growth, blood biochemical constituents, redox status, and humoral immune response of heat-stressed broiler chickens.

## Materials and methods

### Preparation Spirulina platensis aqueous extract

The dried powder of *Spirulina platensis* was supplied by the project entitled “Utilization of marine algae for salt fodders, milk, meat, and fish production under saline conditions,” Desert Research Center of Egypt. The aqueous extract of *S. platensis* was prepared as described by Quoc andDubacq (1997). Briefly, equal amounts of the dried powder of *Spirulina platensis* and distilled water were mixed and shaken well. In an autoclave, *S. platensis* was extracted with water at 120 °C for 1 h. To adjust the pH to 4.0, the citric acid was added to the hot aqueous extract. The hot blend was then centrifuged at 2500 × *g* for 15 min to discard the insoluble fractions and obtain the water-soluble extract. During the experiment, the aqueous extract was prepared as needed to be used fresh.

### Chemical evaluation and antioxidant assays of Spirulina platensis

#### Total phenolic content

The total phenolic contents of dried powder and water extract forms of *S. platensis* were quantified following the Folin-Ciocalteu assay (Kalagatur et al. 2018). A reaction mixture of 150 µl Na_2_CO_3_ (7.5%, w/v) and 150 µl of *S. platensis* aqueous extract or its dried powder suspension was prepared, mixed well, added to 75 µl Folin-Ciocalteu reagent, and diluted ten times with water (1:10, v/v). After a 30-min incubation time, a microtiter plate reader (BioTek Elx808, USA) was used to analyze the mixtures at 750 nm. The results were expressed as Gallic acid equivalent (mg GAE/100 g).

#### Total flavonoid content

The spectrophotometric method was used to quantify the total flavonoid concentration as described by Zhishen et al. (1999). A 2 ml of *S. platensis* aqueous extract or its dried powder suspension and 8 ml distilled deionized water were mixed, and 0.6 ml NaNO_2_ (1:20) was added to the mixture. The solution was vortexed for 5 min, then 1.2 ml AlCl_3_ (1:10) was added. A 4 ml NaOH (1 mol/L) and 4 ml distilled deionized water were added 6 min later. The absorbance was measured at 510 nm after 5 min centrifugation (3300 × *g*) at room temperature, and the results were expressed as Quercetin equivalent (mg QE/100 g).

#### DPPH assay

The antioxidant activity of *S. platensis* aqueous extract or dried powder was estimated by the scavenging 1,1-diphenyl-2-picrylhydrazyl (DPPH˙) radical (Herald et al. 2012). A 1 ml of *S. platensis* sample was mixed with 2 ml ethanolic DPPH. The absorbance was assayed at 517 nm after 30 min of dark incubation. DPPH˙ scavenging activity (%) was estimated as follows:

$$\mathrm{DPPH radical scavenging effect }(\mathrm{\%})=\frac{\mathrm{A}0-\mathrm{A}1}{\mathrm{A}0}\mathrm{x}100$$

where *A*_0_ is the blank absorbance while *A*_1_ is the sample absorbance.

### Total antioxidant capacity

The total antioxidant capacity of *S. platensis* powder or aqueous extract was estimated as described by Prieto et al. (1999). A 1 ml of the sample was added to a 10 ml reagent mixture (28 mM sodium phosphate, 0.6 M sulfuric acid, and 4 mM ammonium molybdate), mixed well, and incubated. After an incubation period of 90 min at 95 °C, samples were kept to cool near room temperature. The sample absorbance was measured against a blank at 695 nm, and the results were expressed as equivalents of ascorbic acid (mg AA/100 g).

### Birds and experimental design

Six hundred 1-day-old Ross 308 male broiler chicks with similar body weight (40.2 ± 0.5 g) were obtained from a commercial hatchery and equally distributed into five experimental groups. Each group contained six replications of 20 chicks each. Birds were housed in a litter floor system with free access to feed and water via manual drinkers and feeders. Chicks were fed a corn-soybean meal-based grower and finisher (1–21 days and 22–35 days) diets (Table 1), which were formulated to meet the nutrients requirements of Aviagen (2014). The experimental groups were divided: The first group (control) fed the basal diet without additives (C). The second and third groups fed the basal diet with 1 g/kg and 2 g/kg *S. platensis* powder (SPP1 and SPP2), while the fourth and fifth groups received treatments of 1 ml/L and 2 ml/L *S. platensis* aqueous extract (SPE1 and SPE2) in the drinking water. All birds were located in the same conditions. Chicks were kept at standard rearing conditions until day 10, and then the ambient temperature was increased from 29 °C to 34 ± 2 °C for 12 h (from 9:00 am to 9:00 pm) for three consecutive days a week until 35 days of age (Abo Ghanima et al. 2020b). Relative humidity ranged from 50 to 60% throughout the experimental period. Broilers were vaccinated orally against Newcastle disease (NDV, Nobilis® ND Clone 30) at 7 and 19 days and Gumboro disease (IBD, Bursine® Plus) at 14 days, and subcutaneously injections against avian influenza and Newcastle disease (H9N1, CEVAC® NEW FLU H9 K) at 7 days. These vaccines were manufactured by MSD animal health Co., Egypt, Zoetis, US, and Ceva Animal Health, Egypt, respectively.

**Table 1:** Feed ingredients and nutrient content of the basal diets

Ingredients	Starter (1–21 days)	Grower (22–35 days)
Maize	55.00	59.00
Soybean meal	38.7	33.4
Soybean oil	2.38	3.90
Limestone	1.17	1.06
Di-calcium phosphate	2.00	1.82
Premix^1^	0.30	0.30
Sodium chloride	0.25	0.25
L-lysine HCl	0.04	0.10
DL-Methionine	0.16	0.17
Calculated composition		
ME (kcal/kg)	2913	3060
CP (%)	22.06	20.15
Calcium (%)	1.04	0.94
Ava. Phosphorus (%)	0.52	0.48
Methionine (%)	0.52	0.50
Lysine (%)	1.30	1.29
TSAA (%)	0.87	0.82

^1^Premix provides per kg of diet: vitamin A, 14,000 IU; vitamin K, 0.004 g; vitamin E, 0.05 g; vitamin D3, 3000 IU; pyridoxine, 0.003 g; cobalamin, 0.006 g; niacin, 0.06 g; pantothenic acid, 0.02 g; choline, 0.15 g; folic acid, 0.0002 g; Ca, 0.048 g; P, 0.00032 g; Mn, 0.1 g; Fe, 0.05 g; Zn, 0.08 g; Cu, 0.01 g; Co, 0.000025 g; iodine, 0.000015 g

### Growth performance and carcass traits

Body weight (BW), weight gain (BWG), and daily feed intake (FI) per replicate were monitored on day 21 and day 35 to estimate the feed conversion ratio (FCR) as (g feed/ g gain). Daily water intake (WI) was also recorded at the same time interval. On day 35, six birds/group (one per replicate) were randomly selected for evaluating carcass traits and collecting blood samples. Dressing (%), carcass yield (%), and relative weight of abdominal fat were calculated. The indices of the immune organs bursa of Fabricius, spleen, and thymus were also calculated as described by Abo Ghanima et al. (2020a).

### Blood biochemical indices

During slaughtering, blood samples were gathered in non-heparinized tubes, kept to clot, and centrifuged at 3000 × *g* for 15 min. Sera samples were separated and stored at -25 °C till the biochemical analyses. Serum concentrations of aspartate and alanine aminotransferases (AST and ALT), alkaline phosphates (ALP), glucose, total proteins (TP), albumin (ALB), creatinine (CR), uric acid (UA), triglycerides (TG), high-density lipoprotein (HDL-cholesterol), low-density lipoprotein (LDL-cholesterol), total cholesterol (TC), and very-low-density lipoprotein (VLDL-cholesterol) were determined spectrophotometrically (Spectronic 1,201, Milton Roy, Ivyland, PA, USA) following the manufacturer’s instructions of the commercial kits (Bio-Diagnostic Co., Egypt). Triiodothyronine hormone (T_3_) was assayed using radioimmunoassay (RIA) kits (Ibrahim et al. 2020).

### Antioxidant activity

Levels of glutathione peroxidase (GPx), malondialdehyde (MDA), and superoxide dismutase (SOD) in sera samples were measured using the ELISA kit of QuantiChrom (BioAssay Systems, USA and Cayman Chemical Company, USA), following the recommended protocol.

### Humoral immunity

Levels of circulating immunoglobulin IgG, IgA, and IgM were evaluated using chicken-specific immunoglobulin ELISA quantitation kits (Bethyl Laboratories Inc., Montgomery, TX, USA). The humoral immune responses against NDV, H9N1, and IBD were estimated using the hemagglutination inhibition test ELISA assay (Indical Bioscience-GmbH, Leipzig, Germany), as described by (Abdel-Moneim et al. 2020a; Elbaz et al. 2021).

### Statistical analysis

All in vitro measurements were performed in triplicate, and data of total antioxidant capacity, total phenols and flavonoids contents, and DPPH assay of dried powder and aqueous extract of *S. platensis* were recorded and analyzed using the independent sample t-test of SPSS package (v 19, SPSS Inc., Chicago, IL, USA). The rest of the measurements were analyzed using the two-way ANOVA as a completely randomized design of the SPSS software procedure. Tukey’s multiple comparison test was used to assess the statistical differences among means at *P* < 0.05.

## Results

### Total phenolic and flavonoid contents and antioxidant activity of S. platensis

The total phenols and flavonoids of *S. platensis* in the powder and aqueous extract forms and their antioxidant activity are shown in Table 2. The aqueous extraction of *S. platensis* reduced (*P* < 0.01) the abovementioned indices compared to the powdered form. Total phenols and flavonoids contents of *S. platensis* powder were 4.9 and 5.2 times higher than that of the aqueous extract, respectively. The DPPH˙ scavenging activity and total antioxidant capacity of *S. platensis* were significantly (*P* < 0.01) declined in the form of the aqueous extract compared to the powdered form (89.11 vs. 57.64, and 4663 vs. 980.4), respectively.

**Table 2:** Total phenolic and flavonoids contents, DPPH assay, and total antioxidant capacity of *Spirulina platensis* in powdered and aqueous extract forms

Items	Powder	Aqueous extract	*P* values
Total phenols (mg GAE/100 g)	1580^a^ ± 63.2	321.3^b^ ± 18.4	< 0.001
Total flavonoids (mg QE/100 g)	176.3^a^ ± 7.65	34.12^b^ ± 5.03	< 0.001
DPPH˙ scavenging activity, %	89.11^a^ ± 5.11	57.64^b^ ± 2.72	< 0.001
Total antioxidant capacity (mg AA/100 g)	4663^a^ ± 117.1	980.4^b^ ± 44.9	< 0.001

*AA* ascorbic acid equivalent, *QE* quercetin equivalent, *DPPH* 1,1-diphenyl-2-picrylhydrazyl, *GAE* gallic acid equivalent

### Growth performance and carcass characteristics

The effects of incorporation levels and forms of *S. platensis* on the growth and carcass traits of Ross 308 male broiler chicks exposed to cyclic heat stress are shown in Table 3. The dietary incorporation of *S. platensis* improved (*P* < 0.01) BW at 21 days, while its inclusion via the drinking water route did not affect the same parameter. At 35 days, final BW was increased (*P* < 0.01) in all treated birds compared with the unsupplemented ones. The highest BW was recorded in SPP2 followed by SPP1. FI and WI did not differ among experimental groups. During the starter (1–21 days) phase, BWG and FCR were improved only in SPP1 and SPP2 groups; however, during the grower (22–35 days) and overall (1–35 days) periods, they were improved in all treated groups compared to the control. The best BWG and FCR were observed in groups fed dietary levels of *S. platensis* followed by those treated via the drinking water route. Dressing percentage and carcass yield were elevated (*P* < 0.01) only in SPP1 and SPP2 compared to the control, while abdominal fat (%) was not affected. The relative weights of the immune organs (bursa of Fabricius, spleen, and thymus) were not significantly affected by dietary and water treatments (Table 6).

**Table 3:** Effect of *Spirulina platensis* in powdered or aqueous extract forms on growth performance of broiler chickens exposed to cyclic heat stress

Indices	Dietary treatments^1^					SEM^2^	Significance levels		
	**C**	**SPP1**	**SPP2**	**SPE1**	**SPE2**		**Level** **effect** **(L)**	**From** **effect** **(F)**	**L × F** **interaction**
**Body weight, g**									
Initial	40.59	40.18	40.68	40.78	40.74	0.033	0.530	0.535	0.830
21 days	863.7^c^	879.5^b^	891.8^a^	862.4^c^	864.7^c^	1.405	< 0.001	< 0.001	< 0.001
35 days	1945.2^d^	1993.5^b^	2020.6^a^	1960.6^c^	1970.1^c^	3.656	< 0.001	< 0.001	< 0.001
**Body weight gain, g.bird.day**^**−1**^									
1–21 days	39.20^c^	39.94^b^	40.53^a^	39.13^c^	39.23^c^	0.067	< 0.001	< 0.001	< 0.001
22–35 days	77.25^d^	79.57^b^	80.63^a^	78.44^c^	78.96^bc^	0.170	< 0.001	< 0.001	0.002
1–35 days	54.42^d^	55.79^b^	56.57^a^	54.85^c^	55.12^c^	0.104	< 0.001	< 0.001	< 0.001
**Water intake, ml.bird.day**^**−1**^									
1–21 days	85.02	84.06	85.52	87.38	85.78	2.074	0.109	0.121	0.091
22–35 days	357.4	353.1	350.2	366.1	361.8	7.163	0.616	0.150	0.176
1–35 days	194.0	191.7	191.4	198.9	196.3	7.097	0.561	0.085	0.073
**Feed intake, g.bird.day**^**−1**^									
1–21 days	47.31	46.72	47.03	46.79	47.30	0.087	0.057	0.508	0.799
22–35 days	161.8	160.1	159.6	160.6	160.2	0.602	0.072	0.631	0.940
1–35 days	93.11	92.10	92.07	92.31	92.46	0.315	0.081	0.518	0.908
**Feed conversion ratio, g feed.g gain**^**−1**^									
1–21 days	1.21^a^	1.17^b^	1.16^b^	1.20^a^	1.21^a^	0.005	< 0.001	< 0.001	< 0.001
22–35 days	2.09^a^	2.01^c^	1.98^d^	2.05^b^	2.03^bc^	0.008	< 0.001	< 0.001	0.006
1–35 days	1.71^a^	1.65^c^	1.63^d^	1.68^b^	1.68^b^	0.007	< 0.001	< 0.001	< 0.001
**Carcass characteristics, %**									
Dressing	70.52^b^	72.11^a^	71.94^a^	70.56^b^	69.96^b^	0.189	< 0.001	< 0.001	< 0.001
Carcass yield	75.70^b^	77.30^a^	77.22^a^	75.88^b^	74.99^b^	0.203	< 0.001	< 0.001	< 0.001
Abdominal fat	0.625	0.589	0.569	0.579	0.602	0.008	0.002	0.292	0.092

^1^Treatment groups: *C* corn-soybean meal-based diet, *SPP1*1 g *Spirulina platensis* powder/kg, *SPP2* 2 g *S. platensis* powder/kg, *SPE1* 1 ml *S. platensis* aqueous extract/L, *SPE2* 2 ml *S. platensis* aqueous extract/L; ^a–d^means with different superscripts are significantly different; ^2^*SEM* standard error of means

### Serum biochemical indices

The effect of *S. platensis* in the powder or aqueous extract forms on blood biochemical indices of broiler chicks exposed to cyclic heat stress are presented in Table 4. Hepatic and renal functions biomarkers (AST, TP, UA, ALB, ALT, and CR) were not affected by the dietary or water treatments except ALP, which was increased in SPE1 compared to the control. Serum glucose and T_3_ concentrations were also non-significantly altered.

**Table 4:** Effect of *Spirulina platensis* in powdered or aqueous extract forms on blood biochemical indices of broiler chickens exposed to cyclic heat stress

Indices	Dietary treatments^1^					SEM^2^	Significance levels		
	**C**	**SPP1**	**SPP2**	**SPE1**	**SPE2**		**Level** **effect** **(L)**	**From** **effect** **(F)**	**L × F** **interaction**
**Protein fractions, g.dl**^**−1**^									
total protein	3.40	3.33	3.26	3.48	3.32	0.025	0.120	0.168	0.456
Albumin	1.53	1.56	1.58	1.61	1.46	0.018	0.277	0.460	0.133
**Liver enzymes activity, U.L**^**−1**^									
AST	68.08	72.14	71.33	62.62	69.75	1.907	0.793	0.358	0.581
ALT	23.74	24.04	22.53	22.69	22.22	0.534	0.597	0.627	0.876
ALP	175.6^bc^	165.1^c^	194.2^ab^	208.0^a^	174.7^bc^	3.504	0.254	0.230	< 0.001
**Renal function biomarkers, mg.dl**^**−1**^									
uric acid	7.08	6.64	6.59	6.82	6.65	0.099	0.157	0.689	0.931
creatinine	0.608	0.591	0.608	0.592	0.617	0.013	0.821	0.916	0.992
**Thyroid activity, ng.ml**^**−1**^									
T3	1.548	1.693	1.429	1.77	1.63	0.037	0.038	0.191	0.505
**Glucose, mg.dl**^**−1**^	248.3	244.8	250.0	230.9	239.4	3.087	0.388	0.198	0.638

^1^Treatment groups: *C* corn-soybean meal-based diet, *SPP1*1 g *Spirulina platensis* powder/kg, *SPP2* 2 g *S. platensis* powder/kg, *SPE1* 1 ml *S. platensis* aqueous extract/L, *SPE2* 2 ml *S. platensis* aqueous extract/L, *AST* aspartate aminotransferase, *ALP* alkaline phosphatase, *ALT* alanine aminotransferase, *T3* triiodothyronine; ^a–c^means with different superscripts are significantly different; ^2^*SEM* standard error of means

### Serum lipid profile

The serum lipid profile of heat-stressed broiler chickens was greatly influenced (*P* < 0.05) by the treatment of *S. platensis* in its powder or aqueous extract forms (Table 5). Serum levels of TG, TC, LDL, and VLDL-cholesterol were decreased in all treated groups except for TC and LDL-cholesterol, which were not altered in SPE2 compared to the control. Blood HDL-cholesterol concentration was elevated (*P* < 0.01) in SPP2 and decreased (*P* < 0.01) in SPE2 compared to the untreated group. Dietary incorporation of *S. platensis* was more effective than its water administration in enhancing the lipid profile of heat-stressed birds.

**Table 5:** Effect of *Spirulina platensis* in powdered or aqueous extract forms on serum lipid profile and antioxidative status of broiler chickens exposed to cyclic heat stress

Indices	Dietary treatments^1^					SEM^2^	Significance levels		
	**C**	**SPP1**	**SPP2**	**SPE1**	**SPE2**		**Level** **effect** **(L)**	**From** **effect** **(F)**	**L × F** **interaction**
**Lipid profile, mg.dl**^**−1**^									
triglycerides	253.5^a^	223.1^c^	226.2^bc^	235.4^b^	227.8^bc^	2.404	< 0.001	0.073	0.015
total cholesterol	219.4^a^	187.9^bc^	194.0^bc^	180.6^c^	206.4^ab^	3.670	< 0.001	0.770	0.381
HDL-cholesterol	46.25^b^	46.46^b^	50.81^a^	45.96^b^	41.84^c^	0.521	0.988	< 0.001	< 0.001
LDL-cholesterol	129.9^a^	97.88^b^	99.22^b^	87.57^b^	119.0^a^	3.410	< 0.001	0.508	0.033
VLDL-cholesterol	50.69^a^	44.62^b^	45.25^bc^	47.07^b^	45.56^bc^	0.481	< 0.001	0.073	0.015
**Antioxidative biomarkers**									
MDA, nmol.ml^−1^	1.053^a^	0.759^b^	0.810^b^	0.875^ab^	1.025^a^	0.030	0.007	0.057	0.034
SOD, U.ml^−1^	128.8^c^	144.8^a^	145.3^a^	136.8^b^	134.9^b^	1.532	0.001	0.538	0.043
GPx, U.ml^−1^	24.88^c^	37.81^a^	34.31^a^	30.50^b^	30.75^b^	0.492	< 0.001	0.104	0.019

^1^Treatment groups: *C* corn-soybean meal-based diet, *SPP1* 1 g *Spirulina platensis* powder/kg, *SPP2* 2 g *S. platensis* powder/kg, *SPE1* 1 ml *S. platensis* aqueous extract/L, *SPE2* 2 ml *S. platensis* aqueous extract/L; *HDL* high-density lipoprotein, *VLDL* very-low-density lipoprotein, *LDL* low-density lipoprotein, *SOD* superoxide dismutase, *MDA* malondialdehyde, *GPx* glutathione peroxidase; ^a–c^means with different superscripts are significantly different; ^2^*SEM* standard error of means

### Serum redox status

The redox status of heat-stressed broiler chickens affected by dietary or water administration of *S. platensis* is presented in Table 5. The incorporation of *S. platensis* in the powder form was superior to the aqueous extract form in enhancing the blood antioxidative biomarkers of birds subjected to cyclic heat stress. Serum MDA concentration was reduced (*P* < 0.05) in SPP1 and SPP2 but did not differ in SPE1 and SPE2. Serum levels of SOD and GPx were elevated in all treated groups compared to the control with the superior to SPP1 and SPP2 groups.

### Humoral immune response

The humoral immune response of broiler chickens treated with *S. platensis* in powdered or aqueous extract forms and exposed to cyclic heat stress is shown in Table 6. Serum level of IgM was increased (*P* < 0.01) in all treated groups, while IgG level and antibody titer against NDV were elevated (*P* < 0.01) only in SPP1 and SPP2 compared to the control. *S. platensis* level, form and their interaction did not affect the circulating IgA level and antibody titers of H9N1 and IBD.

**Table 6:** Effect of *Spirulina platensis* in powdered or aqueous extract forms on humoral immunity and immune organs relative weights of broiler chickens exposed to cyclic heat stress

Indices	Dietary treatments^1^					SEM^2^	Significance levels		
	**C**	**SPP1**	**SPP2**	**SPE1**	**SPE2**		**Level** **effect** **(L)**	**From** **effect** **(F)**	**L × F** **interaction**
**Immunoglobulin (Ig), mg.dl**^**−1**^									
IgM	88.00^c^	200.6^b^	243.5^a^	179.0^b^	177.5^b^	10.44	< 0.001	0.002	0.012
IgG	547.0^c^	818.3^a^	770.5^ab^	687.5^abc^	610.0^bc^	28.99	0.006	0.059	0.383
IgA	263.3	240.7	253.3	241.5	267.0	5.719	0.262	0.682	0.867
**Antibody titers against**									
NDV	6.50^b^	8.00^a^	8.17^a^	7.17^ab^	7.33^ab^	0.181	0.005	0.084	0.458
H9N1	4.67	5.33	5.50	4.83	4.86	0.164	0.435	0.254	0.701
IBD	305.8	335.5	330.2	319.0	316.8	3.489	0.022	0.127	0.536
**Relative weights of immune organs, %**									
Bursa of Fabricius	0.283	0.271	0.279	0.277	0.270	0.006	0.626	0.890	0.775
Spleen	0.253	0.278	0.311	0.269	0.275	0.008	0.001	0.245	0.600
Thymus	0.261	0.303	0.274	0.265	0.270	0.009	0.325	0.264	0.369

^1^Treatment groups: *C* corn-soybean meal-based diet, *SPP1* 1 g *Spirulina platensis* powder/kg, *SPP2* 2 g *S. platensis* powder/kg, *SPE1* 1 ml *S. platensis* aqueous extract/L, *SPE2* 2 ml *S. platensis* aqueous extract/L; *IBD* infectious bursal disease, *H9N1* avian influenza, *NDV* Newcastle disease virus; ^a–c^means with different superscripts are significantly different; ^2^*SEM* standard error of means

## Discussion

*Spirulina platensis*, as an alga, was approved on 16 June 2011 by the commission regulation (EU) No. 575/2011 to be used in animal feeds in its raw, processed, dried, oil, or extract forms. To our knowledge, no earlier studies compared the incorporation impacts of *S. Platensis* in poultry nutrition in the dried or the extract forms, particularly under heat stress conditions. Considering the increase in water consumption and the decrease in voluntary feed consumption of birds in hot climates, the extract form of algae may be more suitable for alleviating the detrimental impact of heat stress on birds. Nevertheless, the extraction techniques differ in efficiency, costs, solvents (water, hexane, or ethanol), and composition and yield of the extract (Michalak et al. 2017). In the present study, we decided to use the hot water extract because most antioxidant constituents and bioactive substances are extracted in water after a pre-treatment of thermal, enzymatic, chemical, or physical cell disruption (Mesalam et al. 2021; Michalak &Chojnacka 2014). Additionally, a mechanical cell disruption was performed to increase the extraction yield and improve the results obtained (Michalak et al. 2017). Deterioration of cell wall structure is required to elaborate its bioactive ingredients such as polysaccharides, polyphenols, cellulose, lignin, minerals, and pigments (Saeid and Chojnacka, 2015). After all the pre-treatments performed in this study, however, the antioxidant activity and total flavonoid and phenolic contents of the aqueous extract of *S. Platensis* were 4 to 5 times lower than that of the dried powder form, which affects its biological activities and impacts on broilers’ productivity and physiological responses.

This study demonstrated that treating heat-stressed birds with *S. platensis*, dried powder improved their growth and feed efficiency better than its aqueous extract. The promoting activity of *S. platensis* of the broilers’ performance and feed efficiency exposed to heat stress has been well-documented (Abdel-Moneim et al. 2021c; Mirzaie et al. 2018). The favorable effect of *S. platensis* could be justified by enhancing the nutrient uptake and balancing the microbial enumeration in the gut due to its content of antibacterial substances such as laminarin and fucoidan (Alwaleed et al. 2021; Khan et al. 2020b; Park et al. 2018). Additionally, the high contents of highly digestible nutrients in *S. platensis* such as carotenoids, proteins (e.g., lectins), essential unsaturated fatty acids, pigments, essential amino acids, and polyphenols exhibit strong antioxidative potential and contribute to improving the health and growth of birds subjected to high ambient temperature (Abdel-Moneim et al. 2021b; Abdel-Moneim et al. 2020d; Shehata et al. 2020; Tavernari et al. 2018). The low potential of *S. platensis* water extract to improve birds’ growth compared to the dried powder form may be due to its low contents of phenols and flavonoids, as we mentioned before. During the extraction, thermal pre-treatment of *S. platensis* may be another cause as it may generate detrimental molecules not appropriate for poultry feeding or induce thermal degradation of some compounds of interest (Starmans and Nijhuis, 1996). Furthermore, the extraction method requires eliminating the insoluble parts of *Spirulina*, which may contain high protein content and active substances that have not been fully extracted.

Carcass traits were positively influenced in broilers received diets with *S. platensis* powder as carcass yield, and dressing (%) was improved. At the same time, these parameters were not affected by the water administration of *S. platensis* extract. Abdominal fat (%) was not altered in all treated groups. These results agree with Moustafa et al. (2021) and Hajati andZaghari (2019), who reported that dietary inclusion of *S. platensis* powder at 0.25, 0.5, and 1% improved carcass traits of broilers reared under hot and thermoneutral conditions. Moreover, Khan et al. (2020a) documented that 0.2% of dietary *S. platensis* increased dressing percentage but did not affect abdominal fat (%). The improvement in carcass traits can be justified by the potential of *S. platensis* powder to provide ample metabolizable energy and nutrients, leading to improved muscle growth and conversion of nutrients to lean meat (Tavernari et al. 2018).

In the present study, both forms of *Spirulina* did not affect renal or hepatic functions of birds exposed to cyclic heat burden. Previous studies reported conflicting results concerning *Spirulina*’*s* impact on hepatic and renal function of experimental animals. Roy-Lachapelle et al. (2017) and Iwasa et al. (2002) reported that *Spirulina* might contain cyanotoxins and microcystin (hepatotoxin), which may disrupt hepatic function. However, Sugiharto et al. (2018) showed no detrimental effect of *Spirulina* on hepatic or renal function biomarkers. In contrast, Moustafa et al. (2021) showed that dietary inclusion of *Spirulina* enhanced liver enzymes activity and renal biomarkers of heat-stressed broilers.

Hypolipidemic activity of *S. platensis* has been documented (Hamza et al. 2020; Mirzaie et al. 2018) and might be attributed to its potential to inhibit cholesterol synthesis and absorption in the gut. Furthermore, the polyphenols content of *S. platensis* may inhibit the pancreatic lipase activity and thereby decrease blood lipid concentration (Abdel-Moneim et al. 2020d; Deng and Chow, 2010). In the current study, treatment of both forms of *S. platensis* reduced the serum concentrations of TG, TC, LDL-cholesterol, and VLDL-cholesterol; however, only SPP2 group was able to elevate blood HDL-cholesterol concentration. Dietary incorporation of *S. platensis* powder was superior in enhancing the lipid profile of heat-stressed birds than administrating the aqueous extract. The findings of Mirzaie et al. (2018), in line with ours revealed a remarkable decrease in TC, TG, and total lipids of broilers fed diets with 2% *S. platensis*. Abdel-Moneim et al. (2021c) also reported an enhancement in the lipid profile of heat-stressed broilers treated with 0.5 and 1% *S. platensis* powder.

Oxidative stress is the major negative impact of heat stress which impairs numerous metabolic dysfunctions and growth performance of broilers (Abo Ghanima et al. 2020a; Liu et al. 2020). GPx and SOD represent the first defense line of the cellular antioxidant mechanism (Ighodaro and Akinloye, 2018), and MDA level indicates the degree of lipid peroxidation in the living cell (Mesalam et al. 2021). The role of dietary supplementation of *S. platensis* to enhance these antioxidative biomarkers of heat-stressed birds has been reported (Abdel-Moneim et al. 2021c; Khan et al. 2020b; Mirzaie et al. 2018; Moustafa et al. 2021). The enhancement in the antioxidant capacity of heat-stressed chicks treated with *S. platensis* might be attributed to its content of antioxidant substances (e.g., phycocyanin, polyphenols, polyunsaturated fatty acids, and β-Carotene) (Pestana et al. 2020). *S. platensis* also has other carotenoids, such as lutein and lycopene, and polysaccharides that characterized for their antioxidant properties (Assunção et al. 2021; Wu et al. 2017). In our study, incorporation of *S. platensis* in the powder form was superior to the aqueous extract form in enhancing the blood antioxidative biomarkers of birds reared under cyclic heat stress. This difference in the antioxidant activity between the powder and aqueous extract forms of *S. platensis* is matched with our in vitro results that revealed a reduction in the total antioxidant capacity, total phenols and flavonoids contents, and DPPH˙ scavenging activity of the aqueous extract of *S. platensis*.

It is well-documented that heat stress impairs humoral immunity and antibody production, particularly after immunization by viral antigens (Hirakawa et al. 2020) and induces inflammatory and oxidative stress (Hirakawa et al. 2020; Song et al. 2018) in heat-stressed broilers. Abdel-Moneim et al. (2020c) and Song et al. (2018) reported a reduction of blood concentrations of immunoglobulin subclasses in heat-stressed birds. Repealing antigen-presenting cells ability to activate T cells might cause the suppression of acquired immunity and antibody production (Preynat-Seauve et al. 2003; Quinteiro-Filho et al. 2010). In the present study, both forms of *S. platensis* increased the serum level of IgM. In contrast, only the powder form elevated serum IgG level and antibody titer against NDV. These findings agree with earlier literature (Khan et al. 2020b; Velten et al. 2018) and confirmed the functional and biological activity of *S. platensis*.

## Conclusion

The present results revealed that incorporating *S. platensis* dried powder or aqueous extract improved the growth and carcass characteristics of broilers reared under cyclic heat stress. The serum lipid profile, redox status, and humoral immune response of these birds were also improved. The powder form of *S. platensis* was superior to the aqueous extract form in improving the studied parameters under cyclic heat stress conditions. Further investigations are required to examine new and more environmentally friendly extraction methods of *S. Platensis*, such as supercritical fluid extraction and microwave-assisted extraction.

## Data Availability

The data used in the present study are available from the corresponding author on reasonable request.

## References

[CR1] Abd El-Hack ME, Alagawany M, Salah AS, Abdel-Latif MA, Farghly MF (2018). Effects of dietary supplementation of zinc oxide and zinc methionine on layer performance, egg quality, and blood serum indices. Biol Trace Elem Res.

[CR2] Abd El-Hack ME, Abdelnour SA, Abd El-Moneim AE, Arif M, Khafaga A, Shaheen H, Samak D, Swelum AA (2019). Putative impacts of phytogenic additives to ameliorate lead toxicity in animal feed. Environ Sci Pollut Res.

[CR3] Abd El-Hack ME, Alagawany M, Abdel-Moneim A-ME, Mohammed NG, Khafaga AF, Bin-Jumah M, Othman SI, Allam AA, Elnesr SS (2020). Cinnamon (Cinnamomum zeylanicum) Oil as a potential alternative to antibiotics in poultry. Antibiotics.

[CR4] Abd El-Hack ME, El-Saadony MT, Shafi ME, Qattan SY, Batiha GE, Khafaga AF, Abdel-Moneim AME, Alagawany M (2020). Probiotics in poultry feed: a comprehensive review. J Anim Physiol Anim Nutr.

[CR5] Abd El-Hack ME, El-Saadony MT, Shafi ME, Alshahrani OA, Saghir SA, Al-Wajeeh AS, Al-Shargi OY, Taha AE, Mesalam NM, Abdel-Moneim A-ME (2021) Prebiotics can restrict Salmonella populations in poultry: a review. Anim Biotechnol 1–10. 10.1080/10495398.2021.1883637 10.1080/10495398.2021.1883637 33607922

[CR6] Abd El-Moneim AE, Sabic EM (2019). Beneficial effect of feeding olive pulp and *Aspergillus awamori* on productive performance, egg quality, serum/yolk cholesterol and oxidative status in laying Japanese quails. J Anim Feed Sci.

[CR7] Abd El-Moneim AE, Sabic EM, Abu-Taleb AM (2019). Influence of dietary supplementation of irradiated or non-irradiated olive pulp on biochemical profile, antioxidant status and immune response of Japanese quails.

[CR8] Abdel-Daim MM, Ahmed A, Ijaz H, Abushouk AI, Ahmed H, Negida A, Aleya L, Bungau SG (2019). Influence of Spirulina platensis and ascorbic acid on amikacin-induced nephrotoxicity in rabbits. Environ Sci Pollut Res.

[CR9] Abdel-Daim MM, Dawood MA, Elbadawy M, Aleya L, Alkahtani S (2020). Spirulina platensis reduced oxidative damage induced by chlorpyrifos toxicity in Nile tilapia (Oreochromis niloticus). Animals.

[CR10] Abdelkhalek NK, Ghazy EW, Abdel-Daim MM (2015). Pharmacodynamic interaction of Spirulina platensis and deltamethrin in freshwater fish Nile tilapia, Oreochromis niloticus: impact on lipid peroxidation and oxidative stress. Environ Sci Pollut Res.

[CR11] Abdel-Latif MA, El-Hack A, Mohamed E, Swelum AA, Saadeldin IM, Elbestawy AR, Shewita RS, Ba-Awadh HA, Alowaimer AN, El-Hamid A (2018). Single and combined effects of Clostridium butyricum and Saccharomyces cerevisiae on growth indices, intestinal health, and immunity of broilers. Animals.

[CR12] Abdel-Latif MA, Elbestawy AR, El-Far AH, Noreldin AE, Emam M, Baty RS, Albadrani GM, Abdel-Daim MM, El-Hamid A, Hatem S (2021). Quercetin dietary supplementation advances growth performance, gut microbiota, and intestinal mrna expression genes in broiler chickens. Animals.

[CR13] Abdel-Moneim A-ME, Sabic E, Abu-Taleb A, Ibrahim N (2020). Growth performance, hemato-biochemical indices, thyroid activity, antioxidant status, and immune response of growing Japanese quail fed diet with full-fat canola seeds. Trop Anim Health Prod.

[CR14] Abdel-Moneim A-ME, Selim DA, Basuony HA, Sabic EM, Saleh AA, Ebeid TA (2020). Effect of dietary supplementation of *Bacillus subtilis* spores on growth performance, oxidative status and digestive enzyme activities in Japanese quail birds. Trop Anim Health Prod.

[CR15] Abdel-Moneim AE, Elbaz AM, Khidr RE, Badri FB (2020). Effect of in ovo inoculation of *Bifidobacterium* spp. on growth performance, thyroid activity, ileum histomorphometry and microbial enumeration of broilers. Probiotics and Antimicrob Proteins.

[CR16] Abdel-Moneim A-ME, El-Saadony MT, Shehata AM, Saad AM, Aldhumri SA, Ouda SM, Mesalam NM (2021a) Antioxidant and antimicrobial activities of Spirulina platensis extracts and biogenic selenium nanoparticles against selected pathogenic bacteria and fungi. Saudi J Biol Sci 29:1197–1209. 10.1016/j.sjbs.2021.09.046 10.1016/j.sjbs.2021.09.046 PMC8848030 35197787

[CR17] Abdel-Moneim A-ME, Shehata AM, Khidr RE, Paswan VK, Ibrahim NS, El-Ghoul AA, Aldhumri SA, Gabr SA, Mesalam NM, Elbaz AM, Elsayed MA, Wakwak MM, Ebeid TA (2021b) Nutritional manipulation to combat heat stress in poultry–a comprehensive review. J Therm Biol 98:102915. 10.1016/j.jtherbio.2021.102915 10.1016/j.jtherbio.2021.102915 34016342

[CR18] Abdel-Moneim A-ME, Shehata AM, Mohamed NG, Elbaz AM, Ibrahim N (2021c) Synergistic effect of Spirulina platensis and selenium nanoparticles on growth performance, serum metabolites, immune responses, and antioxidant capacity of heat-stressed broiler chickens. Biol Trace Elem Res 200:768–779. 10.1007/s12011-021-02662-w 10.1007/s12011-021-02662-w 33674946

[CR19] Abdel-Moneim AME, Shehata AM, Alzahrani SO, Shafi ME, Mesalam NM, Taha AE, Swelum AA, Arif M, Fayyaz M, Abd El-Hack ME (2020). The role of polyphenols in poultry nutrition. J Anim Physiol Anim Nutr.

[CR20] Abedin RM, Taha HM (2008). Antibacterial and antifungal activity of cyanobacteria and green microalgae. Evaluation of medium components by Plackett-Burman design for antimicrobial activity of Spirulina platensis. Glob J Biotechnol Biochem.

[CR21] Abo Ghanima MM, Abd El-Hack ME, Othman SI, Taha AE, Allam AA, Abdel-Moneim A-ME (2020). Impact of different rearing systems on growth, carcass traits, oxidative stress biomarkers and humoral immunity of broilers exposed to heat stress. Poult Sci.

[CR22] Abo Ghanima MM, Bin-Jumah M, Abdel-Moneim A-ME, Khafaga AF, Abd El-Hack ME, Allam AA, El-Kasrawy NI (2020). Impacts of strain variation on response to heat stress and Boldo extract supplementation to broiler chickens. Animals.

[CR23] Abou-Kassem D, Elsadek M, Abdel-Moneim A, Mahgoub S, Elaraby G, Taha A, Elshafie M, Alkhawtani D, Abd El-Hack M, Ashour E (2021). Growth, carcass characteristics, meat quality and microbial aspects of growing quail fed diets enriched with two different types of probiotics (Bacillus toyonensis and Bifidobacterium bifidum). Poult Sci.

[CR24] Agustini T, Suzery M, Sutrisnanto D, Ma’Ruf W,  (2015). Comparative study of bioactive substances extracted from fresh and dried Spirulina sp. Procedia Environ Sci.

[CR25] Aladaileh SH, Khafaga AF, Abd El-Hack ME, Al-Gabri NA, Abukhalil MH, Alfwuaires MA, Bin-Jumah M, Alkahtani S, Abdel-Daim MM, Aleya L, Abdelnour SA (2020) Spirulina platensis ameliorates the sub chronic toxicities of lead in rabbits via anti-oxidative, anti-inflammatory, and immune stimulatory properties. Sci Total Environ 701:134879. 10.1016/j.scitotenv.2019.134879 10.1016/j.scitotenv.2019.134879 31734488

[CR26] Alwaleed E, El-Sheekh M, Abdel-Daim M, Saber H (2021). Effects of Spirulina platensis and Amphora coffeaeformis as dietary supplements on blood biochemical parameters, intestinal microbial population, and productive performance in broiler chickens. Environ Sci Pollut Res.

[CR27] Assunção LS, Bezerra PQM, Poletto VSH, de Oliveira Rios A, Ramos IG, Ribeiro CDF, Machado BAS, Druzian JI, Costa JAV, Nunes IL (2021) Combination of carotenoids from Spirulina and PLA/PLGA or PHB: new options to obtain bioactive nanoparticles. Food Chem 346:128742. 10.1016/j.foodchem.2020.128742 10.1016/j.foodchem.2020.128742 33373823

[CR28] Aviagen R (2014) Ross broiler management handbook. Aviagen Limited Newbridge Midlothian EH28 8SZ Scotland UK. http://pt.aviagen.com/assets/Tech_Center/Ross_Broiler/Ross_Broiler_Manual_9

[CR29] Bogin E, Peh HC, Avidar Y, Israeli BA, Kahaner A (1996). The effects of long term high environmental temperature on cellular enzyme activities from different organs. Clin Chem Lab Med.

[CR30] Deng R, Chow TJ (2010). Hypolipidemic, antioxidant, and antiinflammatory activities of microalgae Spirulina. Cardiovasc Ther.

[CR31] Ding J, He S, Xiong Y, Liu D, Dai S, Hu H (2020) Effects of dietary supplementation of fumaric acid on growth performance, blood hematological and biochemical profile of broiler chickens exposed to chronic heat stress. Brazil J Poult Sci 22. 10.1590/1806-9061-2019-1147

[CR32] Elbaz AM, Ibrahim NS, Shehata AM, Mohamed NG, Abdel-Moneim A-ME (2021). Impact of multi-strain probiotic, citric acid, garlic powder or their combinations on performance, ileal histomorphometry, microbial enumeration and humoral immunity of broiler chickens. Trop Anim Health Prod.

[CR33] Evans A, Smith D, Moritz J (2015). Effects of algae incorporation into broiler starter diet formulations on nutrient digestibility and 3 to 21 d bird performance. J Appl Poult Res.

[CR34] Farag MR, Alagawany M, Abd El-Hack ME, Dhama K (2016). Nutritional and healthical aspects of Spirulina (Arthrospira) for poultry, animals and human. Int J Pharmacol.

[CR35] Hajati H, Zaghari M (2019). Effects of Spirulina platensis on growth performance, carcass characteristics, egg traits and immunity response of Japanese quails. Iran J Appl Anim Sci.

[CR36] Hamza RZ, EL‐Megharbel SM, Altalhi T, Gobouri AA, Alrogi AA (2020) Hypolipidemic and hepatoprotective synergistic effects of selenium nanoparticles and vitamin. E against acrylamide‐induced hepatic alterations in male albino mice. Appl Organomet Chem 34:e5458. 10.1002/aoc.5458

[CR37] Harsini SG, Habibiyan M, Moeini MM, Abdolmohammadi AR (2012). Effects of dietary selenium, vitamin E, and their combination on growth, serum metabolites, and antioxidant defense system in skeletal muscle of broilers under heat stress. Biol Trace Elem Res.

[CR38] Herald TJ, Gadgil P, Tilley M (2012). High-throughput micro plate assays for screening flavonoid content and DPPH-scavenging activity in sorghum bran and flour. J Sci Food Agric.

[CR39] Hirakawa R, Nurjanah S, Furukawa K, Murai A, Kikusato M, Nochi T, Toyomizu M (2020). Heat stress causes immune abnormalities via massive damage to effect proliferation and differentiation of lymphocytes in broiler chickens. Front Vet Sci.

[CR40] Houshmand M, Azhar K, Zulkifli I, Bejo M, Kamyab A (2012). Effects of prebiotic, protein level, and stocking density on performance, immunity, and stress indicators of broilers. Poult Sci.

[CR41] Hu R, He Y, Arowolo MA, Wu S, He J (2019). Polyphenols as potential attenuators of heat stress in poultry production. Antioxidants.

[CR42] Ibrahim N, Sabic E, Abu-Taleb A, Abdel-Moneim A (2020). Effect of dietary supplementation of full-fat canola seeds on productive performance, blood metabolites and antioxidant status of laying Japanese quails. Brazil J Poult Sci.

[CR43] Ighodaro O, Akinloye O (2018). First line defence antioxidants-superoxide dismutase (SOD), catalase (CAT) and glutathione peroxidase (GPX): their fundamental role in the entire antioxidant defence grid. Alex J Med.

[CR44] Iwasa M, Yamamoto M, Tanaka Y, Kaito M, Adachi Y (2002). Spirulina-associated hepatotoxicity. Am J Gastroenterol.

[CR45] Kalagatur NK, Kamasani JR, Mudili V (2018). Assessment of detoxification efficacy of irradiation on zearalenone mycotoxin in various fruit juices by response surface methodology and elucidation of its in-vitro toxicity. Front Microbiol.

[CR46] Khan R, Naz S, Nikousefat Z, Selvaggi M, Laudadio V, Tufarelli V (2012). Effect of ascorbic acid in heat-stressed poultry. Worlds Poult Sci J.

[CR47] Khan S, Mobashar M, Mahsood FK, Javaid S, Abdel-Wareth A, Ammanullah H, Mahmood A (2020). Spirulina inclusion levels in a broiler ration: evaluation of growth performance, gut integrity, and immunity. Trop Anim Health Prod.

[CR48] Liu G, Zhu H, Ma T, Yan Z, Zhang Y, Geng Y, Zhu Y, Shi Y (2020) Effect of chronic cyclic heat stress on the intestinal morphology, oxidative status and cecal bacterial communities in broilers. J Therm Biol 91:102619. 10.1016/j.jtherbio.2020.102619 10.1016/j.jtherbio.2020.102619 32716869

[CR49] Mesalam NM, Aldhumri SA, Gabr SA, Ibrahim MA, Al-Mokaddem AK, Abdel-Moneim A-ME (2021). Putative abrogation impacts of Ajwa seeds on oxidative damage, liver dysfunction and associated complications in rats exposed to carbon tetrachloride. Mol Biol Rep.

[CR50] Michalak I, Chojnacka K (2014). Algal extracts: technology and advances. Eng Life Sci.

[CR51] Michalak I, Chojnacka K, Saeid A (2017). Plant growth biostimulants, dietary feed supplements and cosmetics formulated with supercritical CO2 algal extracts. Molecules.

[CR52] Mirzaie S, Zirak-Khattab F, Hosseini SA, Donyaei-Darian H (2018). Effects of dietary Spirulina on antioxidant status, lipid profile, immune response and performance characteristics of broiler chickens reared under high ambient temperature. Asian-Australas J Anim Sci.

[CR53] Mohamed ASA, Lozovskiy AR, Ali AMA (2020). Nutritional strategies to alleviate heat stress effects through feed restrictions and feed additives (vitamins and minerals) in broilers under summer conditions. J Anim Behaviour and Biometeor.

[CR54] Moustafa ES, Alsanie WF, Gaber A, Kamel NN, Alaqil AA, Abbas AO (2021). Blue-Green Algae (Spirulina platensis) Alleviates the negative impact of heat stress on broiler production performance and redox status. Animals.

[CR55] Park J, Lee S, Kim I (2018). Effect of dietary Spirulina (Arthrospira) platensis on the growth performance, antioxidant enzyme activity, nutrient digestibility, cecal microflora, excreta noxious gas emission, and breast meat quality of broiler chickens. Poult Sci.

[CR56] Pestana J, Puerta B, Santos H, Madeira M, Alfaia C, Lopes P, Pinto R, Lemos J, Fontes C, Lordelo M (2020). Impact of dietary incorporation of Spirulina (Arthrospira platensis) and exogenous enzymes on broiler performance, carcass traits, and meat quality. Poult Sci.

[CR57] Preynat-Seauve O, Coudurier S, Favier A, Marche P-N, Villiers C (2003). Oxidative stress impairs intracellular events involved in antigen processing and presentation to T cells. Cell Stress Chaperones.

[CR58] Prieto P, Pineda M, Aguilar M (1999). Spectrophotometric quantitation of antioxidant capacity through the formation of a phosphomolybdenum complex: specific application to the determination of vitamin E. Anal Biochem.

[CR59] Quinteiro-Filho W, Ribeiro A, Ferraz-de-Paula V, Pinheiro M, Sakai M, Sá L, Ferreira A, Palermo-Neto J (2010). Heat stress impairs performance parameters, induces intestinal injury, and decreases macrophage activity in broiler chickens. Poult Sci.

[CR60] Quoc KP, Dubacq J-P (1997). Effect of growth temperature on the biosynthesis of eukaryotic lipid molecular species by the cyanobacterium Spirulina platensis. Biochim Biophys Acta-Lipids Lipid Metab.

[CR61] Roy-Lachapelle A, Solliec M, Bouchard MF, Sauvé S (2017). Detection of cyanotoxins in algae dietary supplements. Toxins.

[CR62] Saeed M, Naveed M, BiBi J, Kamboh AA, Arain MA, Shah QA, Alagawany M, El-Hack ME, Abdel-Latif MA, Yatoo M (2018). The promising pharmacological effects and therapeutic/medicinal applications of punica granatum L. (Pomegranate) as a functional food in humans and animals. Recent Pat Inflamm Allergy Drug Discov.

[CR63] Saeed M, Naveed M, Leskovec J, Kakar I, Ullah K, Ahmad F, Sharif M, Javaid A, Rauf M, Abd El-Hack ME (2020). Using Guduchi (Tinospora cordifolia) as an eco-friendly feed supplement in human and poultry nutrition. Poult Sci.

[CR64] Saeid A, Chojnacka K (2015) Algae biomass as a raw material for production of algal extracts. Marine Algae Extracts: Processes Prod and Appl 179–188. 10.1002/9783527679577.ch10

[CR65] Sahin K, Sahin N, Kucuk O, Hayirli A, Prasad A (2009). Role of dietary zinc in heat-stressed poultry: a review. Poult Sci.

[CR66] Sakamoto KS, Benincasa NC, da Silva IJO, Lobos CMV (2020). The challenges of animal welfare in modern Brazilian poultry farming. J Anim Behaviour and Biometeorol.

[CR67] Saleh AA, Eltantawy MS, Gawish EM, Younis HH, Amber KA, Abd El-Moneim AE-ME, Ebeid TA (2020). Impact of dietary organic mineral supplementation on reproductive performance, egg quality characteristics, lipid oxidation, ovarian follicular development, and immune response in laying hens under high ambient temperature. Biol Trace Elem Res.

[CR68] Saleh AA, Shukry M, Farrag F, Soliman MM, Abdel-Moneim A-ME (2021). Effect of feeding wet feed or wet feed fermented by Bacillus licheniformis on growth performance, histopathology and growth and lipid metabolism marker genes in broiler chickens. Animals.

[CR69] Shehata AM, Saadeldin IM, Tukur HA, Habashy WS (2020). Modulation of heat-shock proteins mediates chicken cell survival against thermal stress. Animals.

[CR70] Song Z, Cheng K, Zheng X, Ahmad H, Zhang L, Wang T (2018). Effects of dietary supplementation with enzymatically treated Artemisia annua on growth performance, intestinal morphology, digestive enzyme activities, immunity, and antioxidant capacity of heat-stressed broilers. Poult Sci.

[CR71] Starmans DA, Nijhuis HH (1996). Extraction of secondary metabolites from plant material: a review. Trends Food Sci Technol.

[CR72] Sugiharto S (2020). Alleviation of heat stress in broiler chicken using turmeric (Curcuma longa)-a short review. J Anim Behaviour and Biometeorol.

[CR73] Sugiharto S, Yudiarti T, Isroli I, Widiastuti E (2018). Effect of feeding duration of Spirulina platensis on growth performance, haematological parameters, intestinal microbial population and carcass traits of broiler chicks. S Afr J Anim Sci.

[CR74] Surai PF (2007) Natural antioxidants in poultry nutrition: new developments, Proceedings of the 16th European symposium on poultry nutrition. World PoultSci Association, pp 26–30. https://www.feedfood.co.uk/download/1_Europ_Strasbourg_Symposium_2007.pdf

[CR75] Tao X, Xin H (2003). Acute synergistic effects of air temperature, humidity, and velocity on homeostasis of market–size broilers. Trans of the ASAE.

[CR76] Tavernari F, Roza L, Surek D, Sordi C, Silva M, Albino L, Migliorini M, Paiano D, Boiago M (2018). Apparent metabolizable energy and amino acid digestibility of microalgae Spirulina platensis as an ingredient in broiler chicken diets. Br Poult Sci.

[CR77] Velten S, Neumann C, Bleyer M, Gruber-Dujardin E, Hanuszewska M, Przybylska-Gornowicz B, Liebert F (2018). Effects of 50 percent substitution of soybean meal by alternative proteins from Hermetia illucens or Spirulina platensis in meat-type chicken diets with graded amino acid supply. Open J Anim Sci.

[CR78] Wu X, Li R, Zhao Y, Liu Y (2017). Separation of polysaccharides from Spirulina platensis by HSCCC with ethanol-ammonium sulfate ATPS and their antioxidant activities. Carbohydr Polym.

[CR79] Zhishen J, Mengcheng T, Jianming W (1999). The determination of flavonoid contents in mulberry and their scavenging effects on superoxide radicals. Food Chem.

